# Neural correlates of altered interoception in depressive and anxiety disorders: a systematic review and meta-analysis

**DOI:** 10.1007/s00406-026-02201-5

**Published:** 2026-02-07

**Authors:** Lora Bednarek, Ben J. Harrison, Christopher G. Davey, Trevor Steward, Alec J. Jamieson

**Affiliations:** 1 Department of Psychiatry, The University of Melbourne, Victoria Parkville, Australia; 2 Melbourne School of Psychological Sciences, The University of Melbourne, Victoria Parkville, Australia

**Keywords:** Interoception, Depressive disorders, Anxiety disorders, Functional magnetic resonance imaging, Systematic review, Meta-analysis

## Abstract

**Background:**

Interoceptive dysfunction, the impaired sensing and interpretation of internal bodily states, has emerged as a potential transdiagnostic feature across several psychiatric conditions, particularly depressive and anxiety disorders. Despite their high comorbidity, it remains unclear whether these conditions are associated with similar or distinct alterations in brain regions underlying this process. To clarify this, we conducted a systematic review and meta-analysis of functional magnetic resonance imaging studies examining interoceptive processing in depressive and anxiety disorders.

**Methods:**

Using a pre-registered protocol (registration number: CRD42024579900), we conducted systematic searches across five databases: PsycINFO, EMBASE, PubMed, Scopus, and Web of Science. Our initial search yielded 315 unique studies. After applying our inclusion criteria, we identified 11 relevant studies with a total of 659 participants (269 healthy controls and 390 clinical participants).

**Results:**

Our systematic review and meta-analysis revealed significantly reduced activation in the right dorsal mid-insula and left posterior insula during interoceptive tasks in individuals with major depressive disorder. Interestingly, no significant effects were observed for anxiety disorders or the combined sample compared with healthy controls. Qualitative synthesis suggested potential insula hyperactivation in anxiety, aligning with theories of interoceptive hypervigilance.

**Conclusion:**

These findings support models of allostatic dysfunction in depressive and anxiety disorders, highlighting the insula’s role in interpreting bodily signals. Clinically, this underscores the potential of interoception-focused interventions to recalibrate insula processing and alleviate symptoms. Further research in anxiety disorders with reduced methodological heterogeneity would aid in clarifying the effects suggested by the qualitative synthesis.

**Supplementary Information:**

The online version contains supplementary material available at 10.1007/s00406-026-02201-5.

## Introduction

Depressive and anxiety disorders are highly prevalent and impairing mental health conditions. A 2019 global estimate attributed an annual loss of 46.7 million disability-adjusted life years to depressive disorders and 28.7 million to anxiety disorders [1]. These conditions frequently co-occur, with clinical populations showing concurrent anxiety and depression rates ranging from 20 to 67% [2–5]. This comorbidity is associated with more severe illness, greater functional impairments, reduced quality of life, and poorer clinical outcomes [6]. To better characterize this overlap, recent work has attempted to understand the role of transdiagnostic factors involved in the development and maintenance of these disorders, including impaired interoception.

Interoception, or the process of sensing, interpreting, and appraising internal bodily signals, involves all systems critical to the maintenance of homeostasis, such as the cardiovascular, gastrointestinal, thermoregulatory, and respiratory systems [7–9]. Dysfunctional interoception has been proposed to contribute to a variety of symptoms common to depressive and anxiety disorders, including emotional dysregulation, attentional biases, gastrointestinal issues, chronic pain, and sleep disturbances [10–14]. Paulus and Stein [15]conceptualize altered interoception in anxiety and depressive disorders as stemming from altered self-related processing and alliesthesia (i.e., the dependence of the experience of external stimuli on one’s internal state) [15]. Such impaired processing is hypothesized to lead to heightened sensitivity to bodily sensations, which in turn results in amplified emotional reactivity and accompanying withdrawal or avoidance behaviors [15].

Empirical evidence provides partial support for this framework as well as highlighting the complexity of interoceptive dysfunction in anxiety and depressive disorders. Studies have reported alterations in interoceptive accuracy (i.e., the objective ability to monitor internal bodily states), interoceptive sensibility (i.e., subjective measures of one’s confidence in their perceived accuracy or sensitivity to interoceptive signals) and metacognitive interoceptive awareness (i.e., insight into one’s actual interoceptive ability, or the relationship between objective accuracy and subjective beliefs concerning one’s accuracy) across multiple psychiatric disorders, including anxiety and depression [7, 16, 17]. For instance, individuals with major depressive disorder have demonstrated significant impairments in various facets of interoceptive sensibility such as distrust of bodily cues and diminished self-regulation [18]. Moreover, evidence suggests an interaction between the severity of depressive and anxiety symptoms and interoceptive accuracy [19, 20]. Consistent with this inverse relationship between symptom domains, individuals with generalized anxiety disorder have demonstrated greater cardiac interoceptive accuracy than depressed patients as well as healthy controls [21]. Conversely, while individuals with moderately severe major depressive disorder have illustrated cardiac interoceptive accuracy deficits relative to healthy controls, these effects appear to be diminished at higher severity levels [22]. Taken together, these findings highlight heterogeneity of interoceptive functioning across anxiety and depressive symptomatology in a more complex manner than suggested by the Paulus and Stein model.

**Fig. 1:**
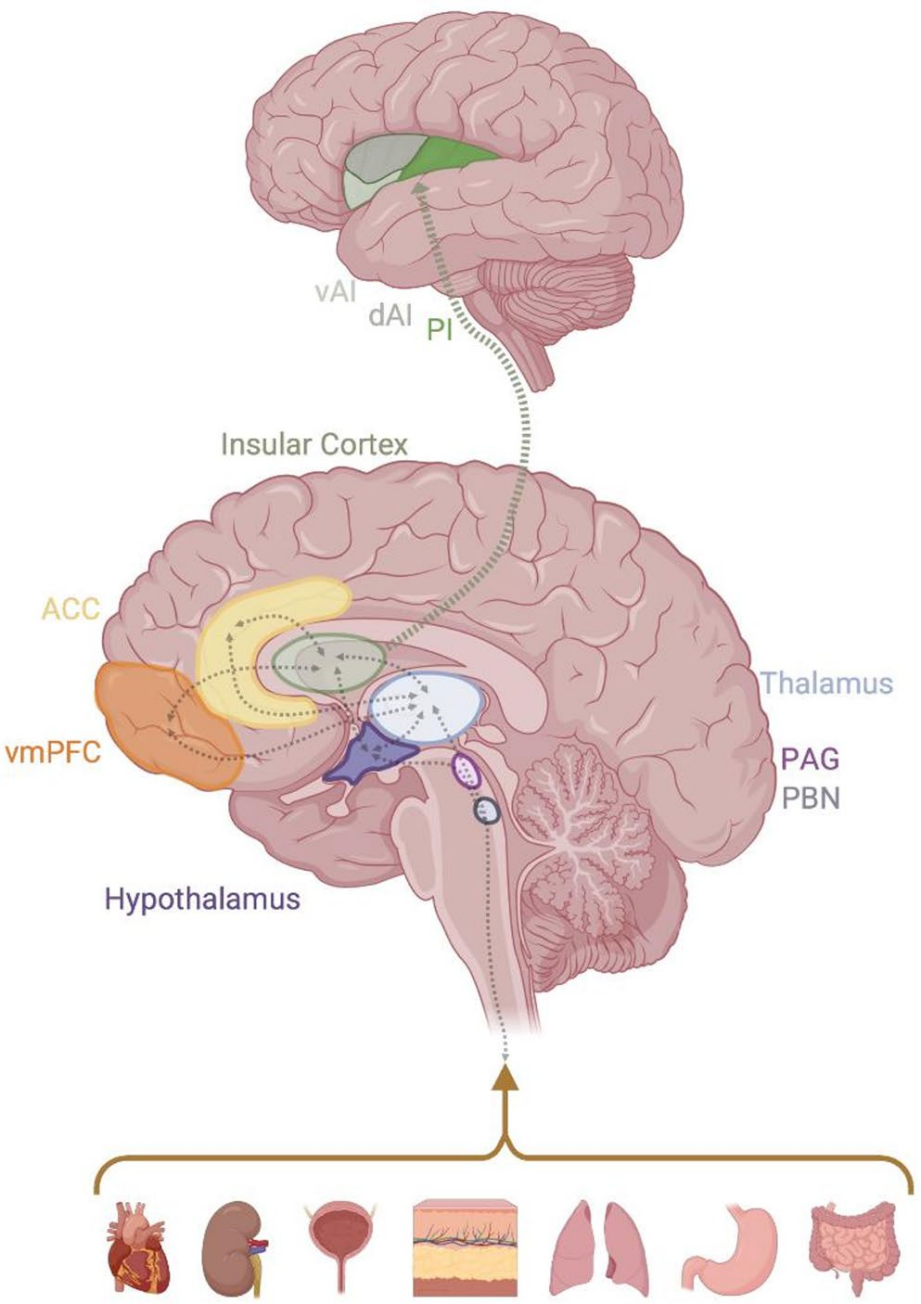
Neural circuitry implicated in interoceptive signal processing. Figure illustrates the key brain regions implicated in the perception and regulation of interoceptive signals—sensations originating from within the body including heart rate, respiration, and bladder fullness. Figure created in BioRender (https://BioRender.com/m6jsd40). *ACC *anterior cingulate cortex,* dAI *dorsal anterior insula*, PAG *periaqueductal gray*, PBN* parabrachial nucleus, *PI* posterior insula, *vAI* ventral anterior insula, *vmPFC* ventromedial prefrontal cortex

Neurobiologically, interoception involves both the peripheral and central nervous system working in tandem to assess the body’s internal state to meet allostatic demands [23, 24]. Peripheral interoceptive signals are transmitted through visceral afferents traveling along both sympathetic and parasympathetic pathways. These signals are integrated in the parabrachial nucleus, which projects extensively to the hypothalamus and periaqueductal gray, demonstrating its role in the maintenance of homeostasis. The integrated signals from the parabrachial nucleus are relayed through the thalamus to various cortical regions including the insular cortex, anterior cingulate cortex (ACC), and ventromedial prefrontal cortex (see Fig. 1 for depiction of key regions). Within this framework, it has been suggested that the dorsal posterior insula primarily processes early interoceptive signals, whereas the anterior insular cortex integrates this information with higher order representations and conscious awareness [23, 25]. The mid-insula appears to have a particular role in interoceptive accuracy, potentially serving as an intermediary between information from the posterior and anterior insula [26, 27]. The ACC is also suggested to play a role in the integration of information, as well as the regulation of homeostasis, supported by a strong connectivity between the anterior insular cortex and ACC [28, 29].

Neuroimaging studies suggest that interoceptive dysfunction in depressive and anxiety disorders may stem primarily from altered insula functioning. Resting-state functional magnetic resonance imaging (fMRI) studies indicate that major depressive disorder is associated with insula hypoactivation, while anxiety disorders show insula hyperactivation [16]. Within the framework of Paulus and Stein, these disruptions may reflect a mismatch between actual interoceptive inputs and predicted bodily states increasing prediction error signals and potentially contributing to interoceptive dysfunction [8, 15]. Supporting this, a meta-analysis by Nord et al. [26] found reduced left dorsal mid-insula activation across psychiatric disorders during interoception tasks compared to healthy controls [26]. Their results suggest that the affective (e.g., anxiety and depressive disorders) and stress-related disorders may be driving this overall effect, but they lacked sufficient data for robust disorder-specific analyses.

The current paper aimed to systematically review fMRI studies of interoception in depressive and anxiety disorders. Given their high comorbidity and overlapping symptomatology, a transdiagnostic analysis may identify shared neural patterns of interoceptive dysfunction across anxiety and depressive disorders. On the other hand, examining these groups independently may reveal disorder-specific trends. Accordingly, neural activation was evaluated in depressive and anxiety samples both collectively and separately. Previous findings highlight the mid-insular cortex as a commonly altered region during interoception across affective disorders [26]. As such, it was hypothesized that both clinical groups would demonstrate altered mid-insula activity during interoceptive tasks compared to controls. However, the direction of this effect remains unclear. Transdiagnostic neuroimaging findings suggest blunted response across disorders [26], while cognitive models of anxiety emphasize heightened bodily sensations [30, 31], indicating the potential for hyperactivation in anxiety disorders.

## Methods

### Search strategy

This review was preregistered with the International Prospective Register of Systematic Reviews (registration number: CRD42024579900) and undertaken in adherence with the Preferred Reporting Items for Systematic-Reviews and Meta-Analyses (PRIMSA; Supplementary Table S1) guidelines [32]. A systematic review of the literature was conducted on the 16th of August 2024 to examine all types of fMRI interoception paradigms used in individuals with depressive and anxiety disorders. We searched for studies of adolescents and adults (> 13 years of age) with a diagnosis of anxiety and/or depression as assessed by a clinically validated diagnostic tool, with healthy adolescents and adults used as comparators or controls. The primary outcome of the systematic review was the differences between clinical and control samples in neural activation during interoceptive tasks (e.g., heartbeat detection, visceral interoceptive attention). We identified studies through searching the electronic databases PsycINFO, EMBASE, PubMed, Scopus, and Web of Science using a combination of key words specifying anxiety and depressive disorders, interoception tasks, and fMRI (see Supplementary Methods for more details) (Table 1).

**Table 1:** Summary of publications investigating interoceptive processing in anxiety and depressive disorders

Author	Year	Clinical population	Sample size	Mean age	Gender % (F)	Clinical diagnosis assessment	Psychotropic medication % (clinical sample)	Interoception task type	Whole Brain or ROI	Interoceptive contrast	Between-group differences (Clinical > HC)
*MDD*											
Burrows	2022	MDD	HC: 41 MDD: 95 (SSRI: 47; Unmed: 48)	HC: 31.5 MDD 34.1	HC: 56.1% MDD 72.7%	MINI (DSM-IV or 5)	49.5%	VIA task (heart & stomach)	Whole Brain and ROI	Interoception vs. exteroception	*Whole Brain* left dorsal mid-insula (↓) bilateral dorsolateral putamen (↓) bilateral dorsal caudate (↓) right amygdala (↓) *ROI* bilateral dorsal anterior insula (↓)
Park	2022	MDD	HC: 27 MDD: 97 (L-RNT: 49, H-RNT: 48)	HC: 31.7 MDD: 34.6	HC: 59.3% MDD: 73.2%	MINI (DSM-IV or 5)	61.9%	VIA task (heart & stomach)	Whole Brain	Interoception vs. exteroception	*Stomach* left medial frontal cortex extending to left insula and operculum (↓) left perirhinal cortex (↓) left caudate nucleus (↓)
DeVille	2018	MDD	HC: 21 MDD: 24	HC: 30.8 MDD: 29.3	HC: 57% MDD: 62.5%	MINI	0%	Breathing interoception	ROI	Interceptive recall vs. exteroceptive recall	right dorsal mid-insula (↓)
Wiebking	2015	MDD	HC: 30 MDD: 22 (current: 12; in remission: 10)	HC: 33.7 MDD: 42	HC: 50% MDD: 50%	DSM-IV criteria	100%	Heartbeat (Critchley and Pollatos)	ROI	Interoception vs. implicit baseline	right dorsal anterior insula (↓) right ventral anterior insula (↓) bilateral posterior insula (↓)
Avery	2014	MDD	HC: 20 MDD: 20	HC: 33 MDD: 36	HC: 60% MDD: 65%	SCID-I (DSM-IV)	0%	VIA task (heart, stomach, bladder)	Whole Brain and ROI	Interoception vs. exteroception	*Heartbeat (Whole Brain)* bilateral dorsal mid-insula (↓) right amygdala (↓) subgenual PFC (↓) lateral OFC (↓) posterior OFC (↓) right caudate nucleus (↓) *Stomach & Bladder (ROI)* bilateral dorsal mid-insula (↓)
Wiebking	2010	MDD	HC: 17 MDD: 17	HC: 37.6 MDD: 41.9	HC: 64.7% MDD: 64.7%	DSM-IV	100%	Heartbeat (Critchley and Pollatos)	ROI	Interoception vs. exteroception	No significant differences
*GAD/PD*											
Teed	2022	GAD	HC: 29 GAD: 29	HC: 24.4 GAD: 26.9	HC: 100% MDD: 100%	MINI (DSM-IV or -5)	20.7%	Pharmacological modulation of cardiorespiratory system	Whole Brain	Peak time vs. baseline time	ventromedial PFC extending to the rostral ACC (↓) left angular gyrus extending into the precuneus (↓)
Cui	2020	GAD	HC: 30 GAD: 32	HC: 31.0 GAD: 33.1	HC: 43.3% GAD: 34.3%	MINI (DSM-IV, Chinese version)	0%	Heartbeat task (Critchley and Pollatos / Wiebking)	Whole Brain	Interoception vs. (a) fixation/(b) exteroception	(a) left anterior insula (↑) (b) right precentral cortex (↑) right anterior insula (↑) left anterior insula (↑) left posterior insula (↑)
Jin	2020	PD	HC: 21 PD: 18	HC: 38.05 PD: 38.11	HC: 62% PD: 66%	MINI (DSM-5, Chinese version)	0%	Heartbeat perception score; Heartbeat task (Critchley and Pollatos / Wiebking)	Whole Brain	Interoception vs. rest-fixation	bilateral SPL (↑) left lingual gyrus (↑) left fusiform gyrus (↑)
McIntosh	2020	PD	HC: 21 PD: 21	HC: 43.6 PD: 43.2	HC: 71.4% PD: 61.9%	SCID (DSM-IV)	0%	Breathing interoception	Whole Brain	Hold vs. Rest	right mid-insula (↑) right precuneus/posterior cingulate (↑) right thalamus (↑) brainstem (↑) bilateral cerebellum (↑) left hippocampus (↑) mid-frontal gyrus (↑) left mid-cingulate cortex (↑) left precentral gyrus (↑) cerebellar vermis (↑)
Goossens	2014	PD	HC: 12 PD: 15	HC: 38.7 PD: 38.3	HC: 41.7% PD: 53.3%	MINI (DSM)	0%	Breathing interoception	ROI	7% CO_2_ vs. 100% O_2_	brainstem (↑)

*MDD* major depressive disorder, *HC* healthy controls, *SSRI* selective serotonin reuptake inhibitor, *MINI* mini-international neuropsychiatric interview, *DSM* diagnostic and statistical manual of mental disorders, *VIA* visceral interceptive attention, *ROI* region of interest, *SCID* structured clinical interview for DSM disorders, *GAD* generalized anxiety disorder, *PD* panic disorder

### Inclusion criteria

**Fig. 2:**
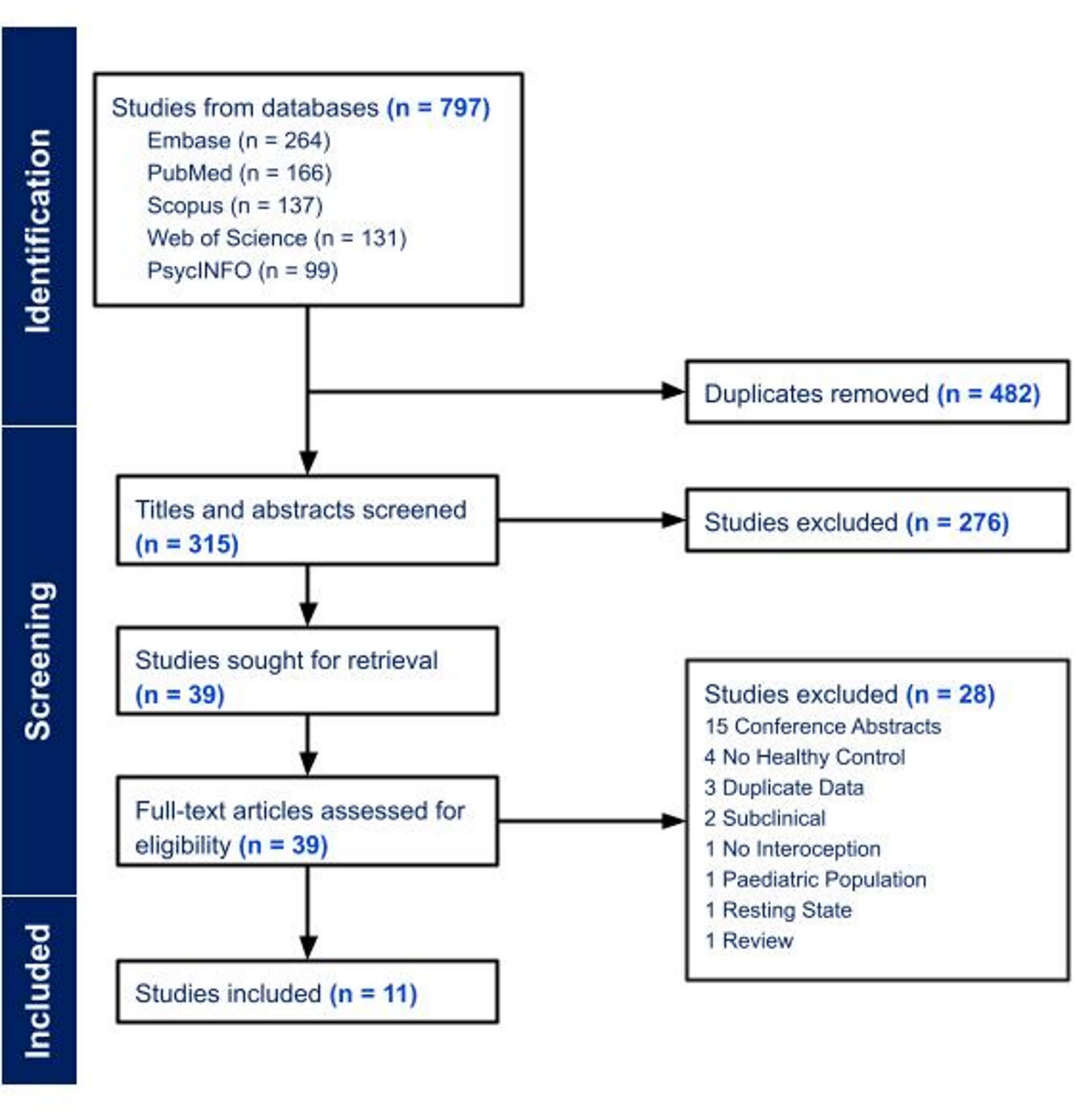
PRISMA flowchart detailing the filtering process for articles

Studies were included if they (a) were peer-reviewed, (b) used an interoception task, (c) included fMRI data, (d) included a patient group with a current or previous diagnosis of a depressive or anxiety disorder assessed through a validated measure, (e) included only human participants, (f) were written in English, and (g) included a control group. Papers that summarized primary studies, such as systematic reviews, or case reports were not included. Studies with subthreshold anxiety and/or depression samples or samples with comorbid neurological or psychiatric diagnoses were excluded. Manual screening of abstracts and full texts using these aforementioned criteria were conducted by two researchers (L.B. and A.J.). Disagreements between reviewers regarding eligibility were resolved by a third reviewer (B.H.). Decisions were organized and recorded using Covidence. See Fig. 2 for the PRISMA flowchart outlining the inclusion process.

### Assessment of risk bias in included studies

The MRI reporting quality assessment criteria was a modified version of the tool reported by Davies et al. [33]. Using these criteria, reported methods were assessed from a range of 0–5 on the following grounds: image acquisition reporting, preprocessing steps reported, statistical modeling and inference, results reporting, and quality checking.

Quality assessment was conducted in the same manner as the manual screening of papers.

### Data extraction and analysis

Data extraction was manually performed independently by two authors (L.B. and A.J.), coding the following variables: first author, year of publication, sample size, type of clinical population, mean age, gender percentage (female), anxiety/depression severity measure, method of classifying anxiety/depression, interoception task type, between-group differences in interoceptive task contrast, and medication status. Due to heterogeneity in the reporting of results, both a qualitative synthesis and meta-analysis were conducted. For details concerning the quality assessment tools used in this review see the Supplementary Materials.

Neurosynth Compose was used to organize the neuroimaging data and to conduct the meta-analyses [34]. A multilevel kernel density (MKDA) meta-analysis [35] was performed with NiMARE0.2.2 (RRID: SCR_017398). A major advantage of MKDA over other methods is that the contrast maps are weighted samples of the study, allowing for a greater contribution from more reliable estimates. An MKDA kernel was used to generate study-wise modeled activation maps from coordinates. In this kernel method, each coordinate is convolved with a sphere with a radius of 10.0 and a value of 1. For voxels with overlapping spheres, the maximum value was retained. Summary statistics (OF values) were converted to p-values using an approximate null distribution. The input dataset included 42 foci from 8 experiments for the full sample, 22 foci from 5 experiments for the depression comparison, and 20 foci from 3 experiments for the anxiety comparison. False discovery rate correction was performed with the Benjamini-Hochberg procedure.

## Results

### Study characteristics

The removal of 482 duplicates from the 797 identified articles resulted in a total of 315 unique papers. Screening of titles and abstracts by two researchers led to 39 papers meeting potential criteria for the review. Full-text screening resulted in 11 papers that met all inclusion criteria. Clinical populations of the studies include major depressive disorder (MDD; six studies), generalized anxiety disorder (GAD; two studies) and panic disorder (PD; three studies). Regarding the interoceptive task, four studies used heartbeat detection and pure tone, three studies used a visceral interoceptive attention (VIA) task, three studies used breathing and/or CO_2_ reactivity, and one study used isoproterenol hydrochloride to modulate the cardiorespiratory system.

Included studies had a combined sample size of 269 healthy controls and 380 clinical participants. The studies had an average sample size of 24 healthy controls (range 12–41) and 35 clinical participants (range 15–97). Within the clinical group, studies had an average sample size of 44 MDD (range 12–97) and 23 GAD/PD (range 15–32). Studies had an average healthy controls age of 34 (range 24.4–43.6) and clinical participant age of 36 (range 26.9–43.2). Within the clinical group, MDD participants had an average age of 36 (range 29.3–42) as did the GAD/PD participants (range 29.6–43.2). Studies had an average gender distribution of 60.5% females in healthy controls (range 41.7–100) and 64% in clinical (range 34.3–100). Within the clinical group, studies had an average of 64.7% female in MDD (range 50-73.2) and 63.1% in GAD/PD (range 34.3–100).

Adherence to the modified COBIDAS guidelines was relatively high among studies, with an average value of 87% (see Supplementary Table S1). Commonly missed items included head coil and echo frequency for acquisition reporting, and type of software used for preprocessing. For two of the studies, employing saline infusion and breathing tasks, experimental design did not meet the criteria in the scale used. In terms of reporting of results, four of the studies only outlined region of interest (ROI) analyses. In both the latter examples, scores of N/A were given.

### Tasks used

*VIA task*: Three studies used variations of the VIA task [36–38]. In short, these tasks required participants to selectively attend to sensations originating from the heart, stomach, and/or bladder. Additionally, this task includes an exteroceptive measure, requiring attention to be directed to an external stimulus, as a control condition.

*Critchley and Pollatos heartbeat task*: This task involved participants undergoing a rest period, interoceptive condition (count heartbeats), and exteroceptive condition (count musical tones). Four studies used variations of this task type [39–42].

*Breathing interoception*: Three of the included studies involved an assessment of interoception associated with respiration [43–45]. One study focused on interoceptive recall. In this study, participants were asked to recall interoceptive or exteroceptive events that they had experienced outside the MRI scanner, while inside the scanner. The other two studies investigated how participants responded to changes in CO_2_ levels in their bodies. One method involved direct detection through being delivered a hypercapnic gas mixture (7% CO_2_), whereas the other used breath-holding.

*Pharmacological modulation of cardiorespiratory system*: Following an infusion of isoproterenol hydrochloride (β-adrenergic receptor agonist), participants were required to continuously rate changes in perceived cardiorespiratory intensity on a 10 point scale [46].

### Narrative synthesis

#### General findings

The most consistent finding was reduced activity in the insular cortex in MDD [36–38, 41, 42]. The effect was observed across multiple task modalities, including VIA, heartbeat, and CO_2_ reactivity, with specific reductions reported in the posterior, dorsal mid- and anterior insula. Conversely, anxiety disorders were characterized by increased activation of the posterior, anterior and mid- insula [39, 45]. Additionally, studies of PD specifically reported increased brainstem activation during CO_2_ reactivity tasks [44, 45]. Other frequently reported regions of altered activation included the dorsal ACC, amygdala, orbitofrontal cortex, hippocampus, and caudate. Despite large variability in the samples and tasks used, only one study reported no differences during interoceptive processing in depressed participants [42]. Generally, larger effects were observed for comparisons between interoceptive and exteroceptive processing, in contrast to comparison between interoceptive processing and rest [39].

#### Whole brain analyses

Three studies utilized a VIA task to examine interoception in MDD with whole-brain analysis [36–38]. All studies reported decreased insula activation in MDD compared to healthy controls. More specifically, Burrows et al. and Avery et al. identified reduced activation in the dorsal mid-insula [36, 37] and Park et al. found reduced activation of the left insula [38]. All three studies reported decreased caudate nucleus activation in MDD. Both Avery et al. and Burrows et al. also found reduced right amygdala activity [36, 37]. Additionally, Park et al. examined rumination subtypes in MDD, finding that individuals with high rumination exhibited diminished hippocampal and amygdala activation compared to those with low rumination [38].

Two studies examined interoception in anxiety disorders using the Critchley and Pollatos heartbeat task [39, 40]. Jin et al. assessed individuals with panic disorder and found increased activation in the bilateral superior parietal lobule in PD compared to controls [40]. Cui et al. studied GAD and reported increased activation in the right precentral cortex and both the anterior and posterior insula in the clinical group [39].

One whole-brain study used a CO_2_ reactivity task in PD [45]. This study identified increased activation in individuals with PD during interoception in many brain regions, including the right mid-insula, right posterior cingulate, right thalamus, bilateral cerebellum, brainstem, and left middle cingulate cortex.

The study which used peripheral adrenergic stimulation revealed reduced activity for GAD participants in the ventromedial prefrontal cortex extending to the rostral ACC as well as in the left angular gyrus extending into the precuneus, though only for the 0.5 μg dose of isoproterenol hydrochloride [46].

#### Region of interest analyses

Two studies used the Critchley and Pollatos heartbeat task for ROI analysis in MDD [41, 42], however, revealed inconsistent findings. Wiebking et al. [42] found no significant differences in interoceptive processing in MDD, whereas Wiebking et al. [41] identified decreased activation in MDD in the right dorsal and ventral anterior insula, as well as the bilateral posterior insula.

Two studies utilized breathing tasks for ROI analysis, one in MDD [43] and the other in PD [44]. DeVille et al. found decreased activity in the right dorsal mid-insula in MDD compared to controls [43]. Conversely, Goossens et al. found increased activation in the brainstem in PD patients compared to controls [44].

### Meta-analysis results

**Fig. 3:**
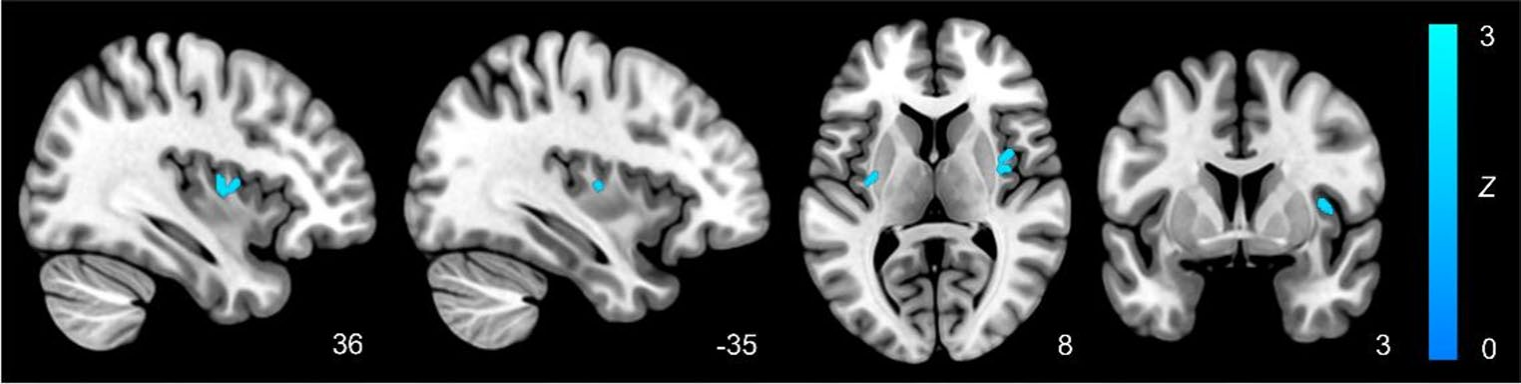
Meta-analysis of interoceptive processing in major depressive disorder participants compared with healthy controls using multilevel kernel density. Results show that those with major depressive disorder demonstrate reduced activation of the dorsal mid- and posterior insular cortex

**Fig. 4:**
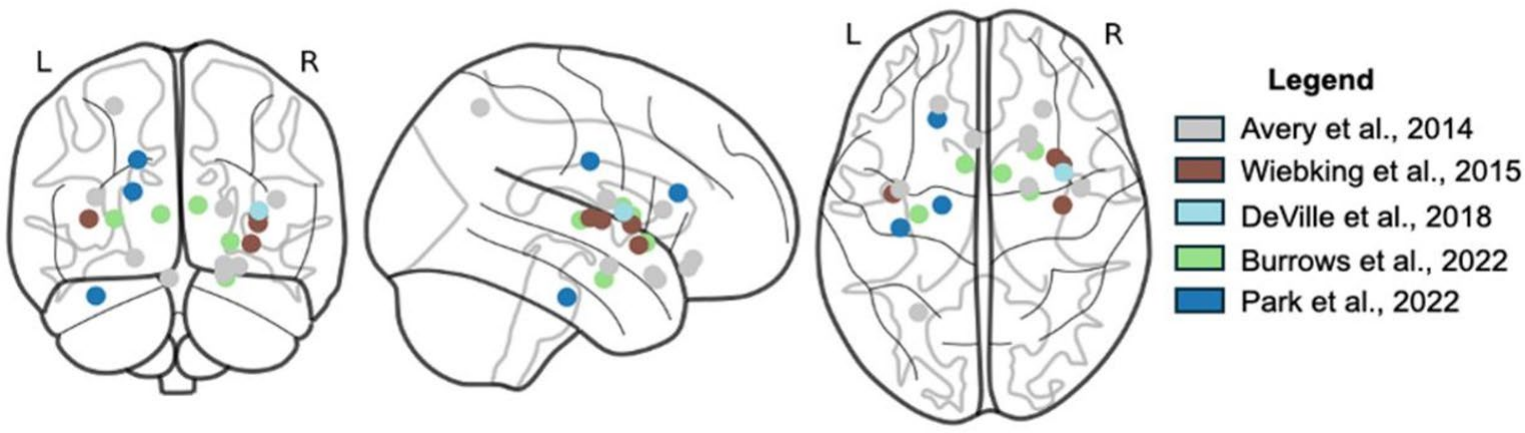
Peak activation coordinates for studies included in the meta-analysis of interoceptive processing alterations in participants with major depressive disorder. Coordinates largely clustered around the mid to anterior as well as posterior insula, with additional peaks being identified across multiple studies in the caudate nucleus and amygdala

From the qualitative sample, sufficient data was reported from eight studies to perform meta-analyses. This included five studies in depressive disorders and three studies in anxiety disorders. Overall, we observed no significant effects for the full sample nor for the anxiety disorder sub-sample. However, when examining MDD alone, there was significantly reduced activation in the right dorsal mid-insula (Z = 2.33; Peak: 38, -4, 8; K = 432mm^3^) as well as the left posterior insula (Z = 2.33; Peak: -34, -10, 8; K = 120mm^3^; Fig. 3). These included results from both whole-brain and ROI analyses and were consistent with the qualitative distribution of coordinates, as observed in Fig. 4.

## Discussion

Our systematic and meta-analytic review examined fMRI studies of interoception in depressive and anxiety disorders. Partially consistent with the primary hypothesis, the meta-analysis confirmed that MDD is characterized by decreased activity compared to controls in the insula, namely the right dorsal mid-insula and left posterior insula. Notably, however, this relationship was not observed for the combined depression and anxiety sample, nor for the anxiety group alone. Qualitative synthesis suggests that anxiety disorders may, in fact, exhibit increased activity in the anterior and mid-insular cortex. However, further work consisting of more homogeneous anxiety studies (both in terms of diagnosis and interoceptive tasks used) will be required to confirm this observation.

The insula has been hypothesized to follow a posterior-to-anterior hierarchy, with primary roles varying from processing afferent signals to gating conscious awareness [47, 48]. This work positions the mid-insula as having a critical role for the integration of information relating to bodily states. Recently, Fermin et al. attempted to expand upon this framework, proposing a three-tiered insula model [49]. The granular insula first receives interoceptive afferent signals and uses these to inform lower-order innate interoceptive representations. Resulting prediction errors are projected to the mid-dorsal insula, which facilitates preemptive adjustments to the visceral system through model-based adaptive behavior, before this information is forwarded onto the agranular insula. As such, the mid-insula has a central role in integrating sensory signals, interoceptive awareness, and prior expectations about the bodily states [50]. Dysfunction across the posterior and mid-insula in MDD participants thus suggests dysfunction in early and model based visceral regulation, prior to conscious awareness and behavioral adaptation facilitated by agranular insula. Moreover, this conceptualization and our insula observations are consistent with findings across several psychiatric disorders [26, 51], supporting the mid-insula as a common locus of disruption. Within depression specifically, it has been hypothesized that reduced mid-insula activation underlies blunted interoceptive processing and thus may contribute to core depressive symptoms of emotional numbing and difficulties in identifying or responding to internal bodily states [8].

One framework that helps contextualize these findings is Barrett et al.’s proposition that depression is a “disorder of allostasis” [52]. Allostasis refers to the body’s ability to anticipate physiological needs in response to external challenges and prepare the body accordingly. Efficient allostatic regulation depends partly on the hypothalamic-pituitary-adrenal (HPA)-axis function, which underpins the body’s hormonal response to stress [53]. Dysregulation of the HPA-axis, as seen in several mental health disorders, can amplify predicted demands and produce inflammatory and oxidative changes (i.e., allostatic overload; [54]). Importantly, effective allostatic regulation depends upon accurate interoceptive processing, which integrates bottom-up somatic signals with top-down cognitive predictions to guide appropriate physiological adjustments. Impairments in interoceptive processing may thus directly contribute to allostatic dysfunction [54]. Given the observed blunted mid-insula activation, it is possible that disrupted interoceptive signaling underlies deficits in both allostatic and homeostatic regulation, contributing to depressive psychopathology. However, the causal relationship between insula dysfunction and depressive symptomatology remains unclear. Insula dysfunction may precede psychopathology via disrupted interoceptive processing or arise as a consequence of chronic stress and trauma that amplify afferent signaling [55]. Mid-insula alterations may represent an attempt to regulate this aberrant afferent signaling, filtering what reaches the higher-level processing and potentially contributing to symptoms of emotional numbing. Though studies have implied a relationship between early traumatic experiences and disturbances in interoceptive accuracy and emotional dysregulation [56], longitudinal studies are needed to clarify the causal nature of these relationships.

Although somatic complaints are not core symptoms within DSM diagnostic criteria, they are emphasized in historic criteria and in phenomenological psychiatry [57, 58]. Anxiety and depression are characterized by pervasive somatic symptoms, including heart palpitations, tingling in the extremities, fatigue, dizziness, and headaches, which often constitute patients’ initial complaints and shape their lived experience with the conditions [56, 58, 59]. The severity of somatic symptoms has been correlated with overall disorder severity in depression and tends to remit alongside affective symptoms, suggesting that interoception represents a central rather than secondary feature of the disorder [59]. Supporting this view, Avery et al. found that insula activity in individuals with MDD was negatively correlated with both affective and somatic symptom severity, highlighting its potential role in linking disrupted bodily state processing to symptom expression [36]. Within this context, the observed insula alterations provide a plausible neural substrate for underlying clinical and phenomenological observations, supporting disrupted interoceptive processing as a core feature to be addressed in the treatment of anxiety and depressive disorders.

The present findings also imply that interoceptive alterations may not reflect a uniform transdiagnostic pattern but instead vary by symptom profile, contrasting the conclusions of Nord et al. [26]. The qualitative findings of overactivation in anxiety disorders implies an opposing effect on insula activation during interoception. It has been hypothesised that heightened insula sensitivity is related to hypervigilance and misinterpretation of benign physiological states, leading to prediction errors and exaggerated stress responses observed in anxiety disorders [15]. Notably, while this relationship was suggested by the qualitative synthesis, it did not reach statistical significance in the meta-analysis, likely due to the use of heterogeneous task design (i.e. the use of respiration and drug intervention design), greater diagnosis variety (i.e. examining panic disorder, social anxiety disorder, and generalized anxiety disorder), and overall fewer anxiety studies. Future work directly comparing functional neuroimaging during interoceptive tasks in anxiety and depressive disorders may help clarify the potential disorder-specific patterns of insula engagement.

Further complicating this relationship is the effect of psychotropic mediations, particularly selective serotonin reuptake inhibitors (SSRIs). SSRIs have been hypothesized to modulate interoceptive processing through both central and peripheral mechanisms, including changes in insula responsivity, visceral sensitivity, and interoceptive sensibility [60]. However, the direction and consistency of these effects remain unclear. For instance, SSRI-medicated MDD participants reported higher intensity ratings of task-related interoceptive sensations compared to unmedicated participants, despite no corresponding differences in brain activation [37]. In contrast, other studies have observed reduced interoceptive abilities in medicated MDD participants, indexed by attenuated emotional experience associated with bodily responses [61]. Further evidence suggests that antidepressants demonstrate limited benefits for some components of interoceptive sensitivity (e.g., decreased distraction of bodily signals) while adversely influencing other interoceptive processes (e.g., self-regulation and body listening) [18]. These findings indicate that SSRIs alter, and may impair, some aspects of interoceptive processing. This represents a limitation of the current literature as medication status was rarely accounted for in analyses or systematically examined, thereby potentially confounding observed effects.

In addition to pharmacological modulation, interoceptive-based psychotherapies also appear to influence this process. Interoceptive exposure therapy, involving systematic exposure to distressing bodily sensations (e.g., chemoreceptive CO_2_ tasks, respiratory or cardiovascular disruptions), has demonstrated efficacy in reducing anxiety symptoms and increasing tolerance of interoceptive signals [62]. Mindfulness-based practices (e.g., yoga and meditation) have been associated with improvements to interoceptive sensibility in anxiety and depression, though the findings remain mixed [63, 64]. Improvements in interoceptive sensibility following these practices are associated with increasing insula responsiveness and reductions in anxiety and depressive symptoms [65–67], highlighting a potential mediatory role. Beyond these approaches, bottom-up interventions that either reduce [68] or increase [69, 70] peripheral multisensory inputs have been highlighted as a potential avenue for treatment of anxiety and depressive disorders, respectively. Although mechanisms to symptom improvement remain unclear, it has been proposed that these therapies recalibrate predictive coding of bodily signals, correcting interoceptive underestimation and insula hypoactivity in depressive disorders [50] and reducing threat-based interpretation of bodily sensations and insula hyperactivity in anxiety disorders [15]. In line with phenomenological psychiatry, these interventions may also be understood as directly addressing core disturbance of embodied self-experience, producing downstream improvements in affective symptoms. Overall, this research places interoceptive processing as a potential marker and treatment target for both depressive and anxiety disorders, offering a promising avenue for precision medicine.

### Limitations

Several limitations should be noted. One limitation is the heterogeneity of interoception task types across studies. Different tasks (e.g., heartbeat detection, breathing, saline infusion) engage differing aspects of interoception and thus likely modulate distinct subregions within the insula [71]. Analyzing evidence from each domain separately, once sufficient data is available, will enable characterization of similarities and differences in neural activation across task types. Related to task variability, commonly used cardiac interoceptive measures, particularly heartbeat perception tasks, show issues concerning validity and reliability. Performance on these tasks appears influenced by external factors such as beliefs about heart rate, time estimation, and task instruction, which may confound observed effects [67, 72]. These psychometric limitations necessitate cautious interpretation of the generalizability of these tasks. Additionally, although the current review initially aimed to examine the relationship between interoceptive sensibility and activation, it was not feasible due to interoceptive sensibility not being routinely assessed. Future research should routinely incorporate measurements of interoceptive awareness, accuracy, and sensibility to clarify the precise nature of this dysfunction and its neural correlates [7, 17]. Another limitation is the restricted consideration of key covariates which have been shown to influence interoceptive processing. Alexithymia is a known risk factor for the development of depressive and anxiety disorders [73] and has been proposed as a mediator between interoceptive dysfunction and the development of depression [74]. Moreover, interoception has been shown to vary among genders at both a behavioral and neural level [75]. Future studies should aim to regularly report as well as adjust for these potentially confounding variables to determine the specificity of interoceptive effects. An additional concern is the small number of studies included, which limits the validity and robustness of conclusions from both qualitative and quantitative analyses. The use of ROI analyses, specifically those including the insula, may have also biased our investigations. Future research with larger sample sizes and whole-brain analysis will aid in addressing these issues.

## Conclusion

Our findings highlight the role of the insula in aberrant interoceptive processing in depressive and anxiety disorders. The meta-analysis revealed that individuals with MDD demonstrated significantly reduced mid-insula activity during interoceptive tasks. Narrative synthesis suggested that those with anxiety disorders may exhibit increased insula activity. However, this was not observed in the meta-analysis likely due to disorder and task heterogeneity as well as the limited sample size. Observed patterns aligned with theories of interoceptive regulation and allostatic dysfunction, suggesting that impaired insula processing contributes to misinterpretation of bodily signals, maladaptive stress responses, as well as potentially contributing to somatic symptoms. Clinically, these insights suggest the utility of interoception-based interventions to potentially recalibrate insula function and reduce symptoms in anxiety and depressive disorders.

## Supplementary Information

Below is the link to the electronic supplementary material.

Supplementary Material 1

## Data Availability

Data extraction for the meta-analysis can be found on Neurosynth Compose: https://compose.neurosynth.org/projects/oieiaeQ8gegG/project.

## References

[1] Global Burden of Disease Collaborative Network (2022) Global, regional, and National burden of 12 mental disorders in 204 countries and territories, 1990–2019: a systematic analysis for the global burden of disease study 2019. Lancet Psychiatry 9:137–150 35026139 10.1016/S2215-0366(21)00395-3 PMC8776563

[2] Eysenck MW, Fajkowska M (2018) Anxiety and depression: toward overlapping and distinctive features. Cogn Emot 32:1391–1400 28608767 10.1080/02699931.2017.1330255

[3] Kalin NH (2020) The critical relationship between anxiety and depression. Am J Psychiatry 177:365–367 32354270 10.1176/appi.ajp.2020.20030305

[4] Koyuncu A, Ertekin E, Binbay Z, Özyıldırım İ, Yüksel Ç, Tükel R (2014) The clinical impact of mood disorder comorbidity on social anxiety disorder. Compr Psychiatry 55:363–369 24262120 10.1016/j.comppsych.2013.08.016

[5] Lamers F, van Oppen P, Comijs HC, Smit JH, Spinhoven P, van Balkom AJLM et al (2011) Comorbidity patterns of anxiety and depressive disorders in a large cohort study: the Netherlands study of depression and anxiety (NESDA). J Clin Psychiatry 72:341–348 21294994 10.4088/JCP.10m06176blu

[6] Roy-Byrne PP, Stang P, Wittchen H-U, Ustun B, Walters EE, Kessler RC (2000) Lifetime panic–depression comorbidity in the National comorbidity survey: association with symptoms, impairment, course and help-seeking. Br J Psychiatry 176(3):229–235 10755069 10.1192/bjp.176.3.229

[7] Garfinkel SN, Manassei MF, Hamilton-Fletcher G (2016) Interoceptive dimensions across cardiac and respiratory axes. Philos Trans R Soc Lond B Biol Sci 371:20160014. 10.1098/rstb.2016.0014 28080971 10.1098/rstb.2016.0014 PMC5062102

[8] Khalsa SS, Adolphs R, Cameron OG, Critchley HD, Davenport PW, Feinstein JS et al (2018) Interoception and mental health: a roadmap. Biol Psychiatry Cogn Neurosci Neuroimaging 3:501–513 29884281 10.1016/j.bpsc.2017.12.004 PMC6054486

[9] Farb N, Daubenmier J, Price CJ, Gard T, Kerr C, Dunn BD et al (2015) Interoception, contemplative practice, and health. Front Psychol 6:763. 10.3389/fpsyg.2015.00763 26106345 10.3389/fpsyg.2015.00763 PMC4460802

[10] Wei Y, van Someren EJ (2020) Interoception relates to sleep and sleep disorders. Curr Opin Behav Sci 33:1–7

[11] Zamariola G, Frost N, van Oost A, Corneille O, Luminet O (2019) Relationship between interoception and emotion regulation: new evidence from mixed methods. J Affect Disord 246:480–485 30599372 10.1016/j.jad.2018.12.101

[12] Zhou H, Zou H, Dai Z, Zhao S, Hua L, Xia Y et al (2022) Interoception dysfunction contributes to the negative emotional bias in major depressive disorder. Front Psychiatry 13:874859 35479498 10.3389/fpsyt.2022.874859 PMC9035634

[13] Ballenger JC (2000) Anxiety and depression: optimizing treatments. Prim Care Companion CNS Disord 2. 10.4088/PCC.v02n0301 10.4088/pcc.v02n0301 PMC181112 15014652

[14] Zbozinek TD, Rose RD, Wolitzky-Taylor KB, Sherbourne C, Sullivan G, Stein MB et al (2012) Diagnostic overlap of generalized anxiety disorder and major depressive disorder in a primary care sample. Depress Anxiety 29:1065–1071 23184657 10.1002/da.22026 PMC3629816

[15] Paulus MP, Stein MB (2010) Interoception in anxiety and depression. Brain Struct Funct 214:451–463 20490545 10.1007/s00429-010-0258-9 PMC2886901

[16] Garfinkel SN, Seth AK, Barrett AB, Suzuki K, Critchley HD (2015) Knowing your own heart: distinguishing interoceptive accuracy from interoceptive awareness. Biol Psychol 104:65–74 25451381 10.1016/j.biopsycho.2014.11.004

[17] Khoury NM, Lutz J, Schuman-Olivier Z (2018) Interoception in psychiatric disorders: a review of randomized, controlled trials with interoception-based interventions. Harv Rev Psychiatry 26:250–263 30188337 10.1097/HRP.0000000000000170 PMC6129986

[18] Zhou H, Liu J, Wu Y, Huang Z, Wang W, Ma Y et al (2024) Unveiling the interoception impairment in various major depressive disorder stages. CNS Neurosci Ther 30:e14923 39154365 10.1111/cns.14923 PMC11330652

[19] Pollatos O, Traut-Mattausch E, Schandry R (2009) Differential effects of anxiety and depression on interoceptive accuracy. Depress Anxiety 26:167–173 19152366 10.1002/da.20504

[20] Dunn BD, Stefanovitch I, Evans D, Oliver C, Hawkins A, Dalgleish T (2010) Can you feel the beat? Interoceptive awareness is an interactive function of anxiety- and depression-specific symptom dimensions. Behav Res Ther 48:1133–1138 20692645 10.1016/j.brat.2010.07.006 PMC2964892

[21] Ehlers A, Breuer P (1992) Increased cardiac awareness in panic disorder. J Abnorm Psychol 101:371–382 1500594 10.1037//0021-843x.101.3.371

[22] Eggart M, Lange A, Binser M, Queri S, Müller-Oerlinghausen B (2019) Major depressive disorder is associated with impaired interoceptive accuracy: a systematic review. Brain Sci 9:131 31174264 10.3390/brainsci9060131 PMC6627769

[23] Craig AD (2003) Interoception: the sense of the physiological condition of the body. Curr Opin Neurobiol 13:500–505 12965300 10.1016/s0959-4388(03)00090-4

[24] Feldman MJ, Bliss-Moreau E, Lindquist KA (2024) The neurobiology of interoception and affect. Trends Cogn Sci 28:643–661 38395706 10.1016/j.tics.2024.01.009 PMC11222051

[25] Kleint NI, Wittchen H-U, Lueken U (2015) Probing the interoceptive network by listening to heartbeats: an fMRI study. PLoS ONE 10:e0133164 26204524 10.1371/journal.pone.0133164 PMC4512728

[26] Nord CL, Lawson RP, Dalgleish T (2021) Disrupted dorsal mid-insula activation during interoception across psychiatric disorders. Am J Psychiatry 178:761–770 34154372 10.1176/appi.ajp.2020.20091340 PMC7613124

[27] Tan Y, Wei D, Zhang M, Yang J, Jelinčić V, Qiu J (2018) The role of mid-insula in the relationship between cardiac interoceptive attention and anxiety: evidence from an fMRI study. Sci Rep 8:17280 30467392 10.1038/s41598-018-35635-6 PMC6250688

[28] Menon V, Uddin LQ (2010) Saliency, switching, attention and control: a network model of Insula function. Brain Struct Funct 214:655–667 20512370 10.1007/s00429-010-0262-0 PMC2899886

[29] Seeley WW (2019) The salience network: a neural system for perceiving and responding to homeostatic demands. J Neurosci 39:9878–9882 31676604 10.1523/JNEUROSCI.1138-17.2019 PMC6978945

[30] Clark DM (1986) A cognitive approach to panic. Behav Res Ther 24:461–470 3741311 10.1016/0005-7967(86)90011-2

[31] Clark DM, Wells A (1995) A cognitive model of social phobia. Social phobia: Diagnosis, Assessment, and treatment. Guilford Press, New York, pp 69–93

[32] Page MJ, McKenzie JE, Bossuyt PM, Boutron I, Hoffmann TC, Mulrow CD, Shamseer L, Tetzlaff JM, Akl EA, Brennan SE, Chou R, Glanville J, Grimshaw JM, Hróbjartsson A, Lalu MM, Li T, Loder EW, Mayo-Wilson E, McDonald S, McGuinness LA, Stewart LA, Thomas J, Tricco AC, Welch VA, Whiting P, Moher D (2021) The PRISMA 2020 statement: an updated guideline for reporting systematic reviews. BMJ 372:71

[33] Davies G, Hayward M, Evans S, Mason O (2020) A systematic review of structural MRI investigations within borderline personality disorder. Psychiatry Res 286:112864 32163818 10.1016/j.psychres.2020.112864

[34] Kent JD, Lee N, Laird AR, Salo T, Peraza J, Bottenhorn KL et al (2026) Neurosynth compose: A Web-Based platform for flexible and reproducible neuroimaging Meta-Analysis. Imaging Neurosci. 10.1162/IMAG.a.1114 10.1162/IMAG.a.1114 PMC12849227 41614048

[35] Wager TD, Lindquist M, Kaplan L (2007) Meta-analysis of functional neuroimaging data: current and future directions. Soc Cogn Affect Neurosci 2:150–158 18985131 10.1093/scan/nsm015 PMC2555451

[36] Avery JA, Drevets WC, Moseman SE, Bodurka J, Barcalow JC, Simmons WK (2014) Major depressive disorder is associated with abnormal interoceptive activity and functional connectivity in the Insula. Biol Psychiatry 76:258–266 24387823 10.1016/j.biopsych.2013.11.027 PMC4048794

[37] Burrows K, DeVille DC, Cosgrove KT, Kuplicki RT, Paulus MP, Aupperle R et al (2022) Impact of serotonergic medication on interoception in major depressive disorder. Biol Psychol 169:108286 35149138 10.1016/j.biopsycho.2022.108286 PMC8958795

[38] Park H, Sanchez SM, Kuplicki R, Tsuchiyagaito A, Khalsa SS, Paulus MP, Guinjoan SM (2022) Attenuated interoceptive processing in individuals with major depressive disorder and high repetitive negative thinking. J Psychiatr Res 156:237–244 36270063 10.1016/j.jpsychires.2022.10.020 PMC11008725

[39] Cui H, Zhang B, Li W, Li H, Pang J, Hu Q et al (2020) Insula shows abnormal task-evoked and resting-state activity in first-episode drug-naïve generalized anxiety disorder. Depress Anxiety 37:632–644 32196828 10.1002/da.23009

[40] Jin H, Zhang B, Cui H, Li W, Li H, Hu Q et al (2020) Altered function of superior parietal lobule associated with perceptive awareness in first-episode drug-naïve panic disorder. Neuropsychiatr Dis Treat 16:1653–1659 32694914 10.2147/NDT.S248453 PMC7340363

[41] Wiebking C, De Greck M, Duncan NW, Tempelmann C, Bajbouj M, Northoff G (2015) Interoception in Insula subregions as a possible state marker for depression. Front Behav Neurosci 9:00082 10.3389/fnbeh.2015.00082 PMC4392695 25914633

[42] Wiebking C, Bauer A, De Greck M, Duncan NW, Tempelmann C, Northoff G (2010) Abnormal body perception and neural activity in the Insula in depression. World J Biol Psychiatry 11:538–549 20146653 10.3109/15622970903563794

[43] DeVille DC, Kerr KL, Avery JA, Burrows K, Bodurka J, Feinstein JS et al (2018) The neural bases of interoceptive encoding and recall in healthy adults and adults with depression. Biol Psychiatry Cogn Neurosci Neuroimaging 3:546–554 29724684 10.1016/j.bpsc.2018.03.010 PMC6415753

[44] Goossens L, Leibold N, Peeters R, Esquivel G, Knuts I, Backes W et al (2014) Brainstem response to hypercapnia: a symptom provocation study into the pathophysiology of panic disorder. J Psychopharmacol (Oxf) 28:449–456 10.1177/0269881114527363 24646808

[45] McIntosh RC, Hoshi RA, Timpano KR (2020) Take my breath away: neural activation at breath-hold differentiates individuals with panic disorder from healthy controls. Respir Physiol Neurobiol 277:103427 32120012 10.1016/j.resp.2020.103427

[46] Teed AR, Feinstein JS, Puhl M, Lapidus RC, Upshaw V, Kuplicki RT et al (2022) Association of generalized anxiety disorder with autonomic hypersensitivity and blunted ventromedial prefrontal cortex activity during peripheral adrenergic stimulation: a randomized clinical trial. JAMA Psychiatry 79:323–332 35107563 10.1001/jamapsychiatry.2021.4225 PMC8811711

[47] Craig AD (2009) How do you feel—now? The anterior Insula and human awareness. Nat Rev Neurosci 10:59–70 19096369 10.1038/nrn2555

[48] Gu X, Hof PR, Friston KJ, Fan J (2013) Anterior insular cortex and emotional awareness. J Comp Neurol 521:3371–3388. 10.1002/cne.23368 23749500 10.1002/cne.23368 PMC3999437

[49] Fermin ASR, Friston K, Yamawaki S (2022) Insula interoception, active inference and feeling representation. R Soc Open Sci 29:220226 10.1098/rsos.220226 PMC9240682 35774133

[50] Barrett LF, Simmons WK (2015) Interoceptive predictions in the brain. Nat Rev Neurosci 16:419–429 26016744 10.1038/nrn3950 PMC4731102

[51] Jamieson AJ, Davey CG, Pujol J, Blanco-Hinojo L, Harrison BJ (2025) Graded changes in local functional connectivity of the cerebral cortex in young people with depression. Psychol Med 55:e88 40091390 10.1017/S0033291725000510 PMC12080650

[52] Barrett LF, Quigley KS, Hamilton P (2016) An active inference theory of allostasis and interoception in depression. Philos Trans R Soc B Biol Sci 371:20160011 10.1098/rstb.2016.0011 PMC5062100 28080969

[53] Bonaz B, Lane RD, Oshinsky ML, Kenny PJ, Sinha R, Mayer EA, Critchley HD (2021) Diseases, disorders, and comorbidities of interoception. Trends Neurosci 44:39–51 33378656 10.1016/j.tins.2020.09.009

[54] Santamaría-García H, Migeot J, Medel V, Hazelton JL, Teckentrup V, Romero-Ortuno R et al (2025) Allostatic interoceptive overload across psychiatric and neurological conditions. Biol Psychiatry 97:28–40 38964530 10.1016/j.biopsych.2024.06.024 PMC12012852

[55] Schaan VK, Schulz A, Rubel JA, Bernstein M, Domes G, Schächinger H, Vögele C (2019) Childhood trauma affects stress-related interoceptive accuracy. Front Psychiatry 10:750 31681049 10.3389/fpsyt.2019.00750 PMC6813623

[56] Medford N, Quadt L, Critchley H (2024) Interoception and psychopathology. In: Mishara AL, Moskalewicz M, Schwartz MA, Kranjec A (eds) Phenomenological neuropsychiatry: how patient experience bridges the clinic with clinical neuroscience. Springer, Cham, pp 155–174

[57] Kendler KS (2016) The phenomenology of major depression and the representativeness and nature of DSM criteria. Am J Psychiatry 173:771–780 27138588 10.1176/appi.ajp.2016.15121509

[58] Domschke K, Stevens S, Pfleiderer B, Gerlach AL (2010) Interoceptive sensitivity in anxiety and anxiety disorders: an overview and integration of Neurobiological findings. Clin Psychol Rev 30:1–11 19751958 10.1016/j.cpr.2009.08.008

[59] Harshaw C (2015) Interoceptive dysfunction: toward an integrated framework for Understanding somatic and affective disturbance in depression. Psychol Bull 141:311–363 25365763 10.1037/a0038101 PMC4346391

[60] Nord CL, Garfinkel SN (2022) Interoceptive pathways to understand and treat mental health conditions. Trends Cogn Sci 26:499–513 35466044 10.1016/j.tics.2022.03.004

[61] Lyons N, Strasser A, Beitz B, Teismann T, Ostermann T, Anderle L, Michalak J (2021) Bodily maps of emotion in major depressive disorder. Cogn Ther Res 45:508–516

[62] Schoeller F, Horowitz AH, Jain A, Maes P, Reggente N, Christov-Moore L et al (2024) Interoceptive technologies for psychiatric interventions: from diagnosis to clinical applications. Neurosci Biobehav Rev 156:105478 38007168 10.1016/j.neubiorev.2023.105478

[63] Khalsa SS, Rudrauf D, Hassanpour MS, Davidson RJ, Tranel D (2020) The practice of meditation is not associated with improved interoceptive awareness of the heartbeat. Psychophysiology 57:e13479 31573689 10.1111/psyp.13479 PMC6982546

[64] Heim N, Bobou M, Tanzer M, Jenkinson PM, Steinert C, Fotopoulou A (2023) Psychological interventions for interoception in mental health disorders: a systematic review of randomized-controlled trials. Psychiatry Clin Neurosci 77:530–540 37421414 10.1111/pcn.13576/full PMC7615164

[65] Datko M, Lutz J, Gawande R, Comeau A, To MN, Desel T et al (2022) Increased Insula response to interoceptive attention following mindfulness training is associated with increased body trusting among patients with depression. Psychiatry Res Neuroimaging 327:111559 36308976 10.1016/j.pscychresns.2022.111559 PMC12981234

[66] Gonsalves M, Beck Q, Fukuda A, Tirrell E, Kokdere F, Kronenberg E et al (2021) Mechanical affective touch therapy (MATT) for anxiety disorders: effects on resting-state functional connectivity. Brain Stimul 14:1630–1631 10.1016/j.neurom.2021.10.007 PMC9256848 35088729

[67] Jenkinson PM, Fotopoulou A, Ibañez A, Rossell S (2024) Interoception in anxiety, depression, and psychosis: a review. eClinicalMedicine 73:102673 38873633 10.1016/j.eclinm.2024.102673 PMC11169962

[68] Feinstein JS, Khalsa SS, Yeh H, Al Zoubi O, Arevian AC, Wohlrab C et al (2018) The elicitation of relaxation and interoceptive awareness using floatation therapy in individuals with high anxiety sensitivity. Interoception Ment Health 3:555–562 10.1016/j.bpsc.2018.02.005 PMC6040829 29656950

[69] Canbeyli R (2013) Sensorimotor modulation of mood and depression: in search of an optimal mode of stimulation. Front Hum Neurosci 7:428. 10.3389/fnhum.2013.00428 23908624 10.3389/fnhum.2013.00428 PMC3727046

[70] Canbeyli R (2022) Sensory stimulation via the visual, auditory, olfactory and gustatory systems can modulate mood and depression. Eur J Neurosci 55:244–263 34708453 10.1111/ejn.15507

[71] Critchley HD, Harrison NA (2013) Visceral influences on brain and behavior. Neuron 77:624–638 23439117 10.1016/j.neuron.2013.02.008

[72] Murphy J, Brewer R, Hobson H, Catmur C, Bird G (2018) Is alexithymia characterised by impaired interoception? Further evidence, the importance of control variables, and the problems with the heartbeat counting task. Biol Psychol 136:189–197 29803614 10.1016/j.biopsycho.2018.05.010

[73] Kieraitė M, Bättig JJ, Novoselac A, Noboa V, Seifritz E, Rufer M et al (2024) Our similarities are different: the relationship between alexithymia and depression. Psychiatry Res 340:116099 39173349 10.1016/j.psychres.2024.116099

[74] Brewer R, Cook R, Bird G (2016) Alexithymia: a general deficit of interoception. R Soc Open Sci 3:150664 27853532 10.1098/rsos.150664 PMC5098957

[75] Longarzo M, Mele G, Alfano V, Salvatore M, Cavaliere C (2021) Gender brain structural differences and interoception. Front Neurosci 14:586860 33488344 10.3389/fnins.2020.586860 PMC7815642

